# Evaluation of Serological Tests for Different Disease Stages of Leptospirosis Infection in Humans

**DOI:** 10.3390/tropicalmed9110283

**Published:** 2024-11-20

**Authors:** Virginia C. Rodríguez-Rodriguez, Ana María Castro, Ronald Soto-Florez, Luis Urango-Gallego, Alfonso Calderón-Rangel, Piedad Agudelo-Flórez, Fernando P. Monroy

**Affiliations:** 1 Grupo de Investigaciones Microbiológicas y Biomédicas de Córdoba (GIMBIC), Universidad de Córdoba, Córdoba 230002, Colombia; vrodriguez@correo.unicordoba.edu.co (V.C.R.-R.); anacastroc@correo.unicordoba.edu.co (A.M.C.); soto-36@hotmail.com (R.S.-F.); ferchourango@hotmail.com (L.U.-G.); 2 Instituto de Investigaciones Biológicas del Trópico (IIBT), Universidad de Córdoba, Córdoba 230002, Colombia; acalderonr@correo.unicordoba.edu.co; 3 Ciencias de la Vida y la Salud, Escuela de Graduados, Universidad CES, Medellín 050021, Colombia; pagudelo@ces.edu.co; 4 Department of Biological Sciences, Northern Arizona University, Flagstaff, AZ 86001, USA; 5 Pathogen and Microbiome Institute, Northern Arizona University, Flagstaff, AZ 86011, USA

**Keywords:** Colombia, diagnosis, serology, seroprevalence, convalescent, ELISA, MAT

## Abstract

Background/Objectives: Leptospirosis is a zoonotic disease that is widely distributed around the world and presents symptoms similar to other febrile illnesses in tropical regions, which complicates clinical diagnosis. This study aimed to evaluate the performance and agreement between serological diagnostic tests for detecting both acute and convalescent human leptospirosis, using the micro agglutination test (MAT) as a reference in an endemic region of the Colombian Caribbean. Methods: A prospective descriptive study was conducted on 275 participants with suspected leptospirosis. Paired serum samples were obtained, and an epidemiological survey was conducted. Using the MAT as the gold standard, we calculated positive and negative predictive values, sensitivity, specificity, and kappa index. A Bayesian latent class model was also used to compare the diagnostic tests. Results: In 223 paired serum samples, the sensitivity values for various stages of the disease ranged between 10.8% to 54.1% in the acute and 6.1% to 66.7% during the convalescent phase compared to the MAT. According to the Bayesian model, sensitivity was 9.5% to 75.3% in the acute phase and 5.7% to 85.3% in the convalescent phase. The Kappa value, an indicator of agreement, was moderate for the IgM ELISA in the acute phase (0.553) and substantial in the convalescent phase (0.692). Conclusions: The MAT was the best confirmatory test in both acute and convalescent phases of leptospirosis. Despite the high specificity of ELISA, 21.62% of participants identified as negative by IgM-ELISA in both phases were subsequently confirmed as positive by the MAT. It is necessary to re-evaluate diagnostic guidelines that do not employ the MAT for confirmation and to enhance the diagnostic and clinical identification of leptospirosis within healthcare institutions and public health laboratories while providing a rapid and reliable test for its implementation.

## 1. Introduction

Leptospirosis is a zoonosis with worldwide distribution and a rise in cases associated with increased rainfall and high temperatures. However, cases can occur at any time of the year [1], and it is traditionally considered an occupational exposure disease [2,3,4]. Leptospirosis is notifiable in some countries; however, in general, it is under-reported due to a lack of knowledge of the disease, its similarity with other febrile diseases present in endemic areas, and difficulties in its clinical and laboratory diagnosis [5,6,7]. Laboratory diagnosis allows for the confirmation of leptospirosis where the disease is suspected based on clinical aspects, further determining the serovar causing the infection, the probable source of infection, and the potential reservoir and its location. This information contributes to the implementation of control strategies [8].

Clinical leptospirosis is a biphasic disease, with an acute phase between the 4th and 10th day of disease onset and a convalescent phase ranging from 4 to 30 days [9]. During the acute phase, bacteria are present in the blood, while in the convalescent phase, they disappear from the blood with the appearance of IgM antibodies [10]. Several methods are used for the laboratory diagnosis of leptospirosis: the microscopic agglutination test (MAT), detection of the organism’s DNA by polymerase chain reaction (PCR), the isolation of the microorganism by culture methods, or the detection of antibodies against the microorganism [11]. For many years, serological diagnosis has been considered the cornerstone for identifying leptospiral infections. Typically, these studies are based on detecting specific antibodies against various leptospiral antigens [12]. The isolation of *Leptospira* spp. from clinical samples has low diagnostic sensitivity and requires experienced personnel, and, most importantly, culturing leptospires takes weeks. Therefore, the diagnosis of leptospirosis relies on serological results [12].

During the acute phase of leptospirosis, timely confirmation is an essential clinical priority to optimize targeted treatment and supportive management [13]. Because of their ease of use and comparable sensitivity and specificity, serological tests such as ELISA and rapid lateral flow assays have largely replaced the traditional MAT, especially during the acute stage of the disease [14]. Some of these serological tests now serve as rapid point-of-care screening tests [15]. ELISA has been widely used to detect IgM. However, its specificity is affected by the antigen used in the test, the presence of antibodies from previous exposure (in endemic regions), and other febrile diseases [1]. IgM detection tests have been developed in various rapid assay formats (dipstick, latex agglutination, lateral flow, and bidirectional platform) for implementation in the field or rural clinical laboratories [12]. However, there are significant limitations to early diagnosis using any serological tests, and when performing them, it should be mandatory to use paired sera [16]. Furthermore, it has been recommended that rapid diagnostic tests confirm results using a reference test [15].

The MAT detects both IgM and IgG and is regarded as the gold standard for diagnosing leptospirosis. A confirmed case of leptospirosis should have an acute MAT titer of ≥1 in 400 or a four-fold rise in titer between acute and convalescent samples. Furthermore, the MAT requires a high level of technical experience and the precise time of sample collection [5]. It also requires the maintenance of a diverse live panel of serovars from different serogroups of pathogenic leptospires [1]. These live pathogens can create a risk of laboratory-acquired infections, making them poorly accessible for conventional clinical laboratories [17]. The MAT can also produce many false negative results in the early stage of infection since IgM antibodies detected by this test appear after day eight of infection and reach their peak in the fourth week, with detectable serovar-specific antibody titers persisting for several months and even years [18,19]. Thus, cross-reactions between serogroups occur mainly in the early stages of the disease [20]. Although this test is highly specific, it has limited sensitivity in the acute phase because *Leptospira* antibodies are detectable around 7–10 days after the appearance of symptoms, and commonly, a second serum sample is required for case confirmation, delaying diagnosis and treatment [21,22]. The World Health Organization (WHO) recommends using a locally optimized MAT panel containing strains circulating in a particular study region [8]. The basis for this is to improve the test’s sensitivity since participants’ sera are likely to react well with local strains [14]. However, knowledge about circulating strains is scarce in many highly endemic regions. Even the use of strains representative of a broad panel of serogroups for the MAT is not feasible, given the considerable resources needed for its implementation and the cost of the procedure [14,23]. To date, in Colombia, native strains are not included in the diagnostic panels of the national surveillance system, which could improve the performance of the MAT.

The MAT is a valuable serological reference test for epidemiologic studies and has been considered an imperfect standard for comparing rapid diagnostic evaluation tests [24]. The Bayesian latent class model acknowledges that all tests are imperfect and has been proposed as a more suitable method for evaluating diagnostic tests, including immunodiagnostics for leptospirosis [24,25,26]. This study aimed to analyze the performance and agreement of four serological diagnostic tests available for detecting acute and convalescent human leptospirosis compared with the MAT in an endemic region of the Colombian Caribbean. Our results should provide rural health clinics and diagnostic laboratories that cannot implement the MAT with a reliable alternative to detecting serum samples from suspected cases of leptospirosis.

## 2. Materials and Methods

### 2.1. Ethical Considerations

Subjects were invited to participate in the project. After ensuring eligibility, they were provided with an informed consent form (approved by the ethics committee of the Faculty of Health Sciences of the University of Córdoba). The study was explained to them, and an epidemiological survey was provided after signing the consent form. The Institutional Ethics Committee of the Faculty of Health Sciences considered the study as risk-free according to the technical, scientific, and administrative standards for health research of the Colombian Ministry of Health (Resolution 008430, 4 October 1993) and the Declaration of Helsinki [27].

### 2.2. Operational Definition of Suspicious Case

A suspicious case is defined as a patient who has a fever or a history of fever (>38 °C) in the last three weeks who presents two or more of the following symptoms: headache, myalgia, conjunctivitis, arthralgia, vomiting, diarrhea, back pain, chills, retroocular pain or photophobia, and possibly a rash. Additionally, one or more of these manifestations may indicate disease progression with organ involvement, notably jaundice, liver failure, kidney failure, hematological disorders, or encephalopathy. A suggestive epidemiological history that raises suspicion includes exposure to floods, mud, or stagnant water, whether through work or recreational activities. This may also include occupational risks such as garbage collection, stream cleaning, working in water or wastewater, engaging in agricultural activities, and contact with sick animals or rodents [28].

### 2.3. Study Design

We performed a cross-sectional study at the Department of Córdoba. A total of 275 participants who attended three healthcare institutions were recruited between December 2017 and March 2020 and met the operational case definition established by the Colombian National Institute of Health (INS) [28]. The Department of Córdoba residents were recruited through an alliance with the Department of Public Health laboratory. We excluded participants who did not meet the operational case definition and those who received antibiotic therapy before sample collection. We applied a structured questionnaire to provide information on the individual characteristics of the participants, domestic and peri-domestic environmental characteristics, exposure to sources of environmental contamination, and the presence of potential animal reservoirs.

### 2.4. Collection and Processing of Samples

Blood serum samples were collected from eligible individuals in tubes without additives. All the samples were stored at room temperature and processed within 2 h. Whole-blood samples were collected during the acute and convalescent phases. The time period between sampling for paired sera was 10 and 15 days. Blood samples were transported at 4 °C to the research laboratory of the Microbiological and Biomedical Research Group of Córdoba (GIMBIC), Bacteriology Program, University of Cordoba.

Paired sera were analyzed using the commercial kit Panbio^TM^ Leptospira IgM ELISA, immunochromatography (IgM) was performed using two commercial kits (SD Bioline Leptospira IgM^TM^ and Leptocheck WB^TM^) according to the manufacturer’s instructions, and the microagglutination test (MAT) was performed according to WHO specifications [8], using 14 serogroups and 19 serovars available in the GIMBIC laboratory from the research group with 14 serogroups: *Australis* serovar *Australis*, Bratislava; serogroup *Autumnalis* serovar *Autumnalis*, serogroup *Ballum* serovar *Ballum*, serogroup *Bataviae* serovar *Bataviae*, serogroup *Canicola* serovar *Canicola*, serogroup *Celledoni* serovar *Celledoni*, serogroup *Grippotyphosa* serovar *Grippotyphosa*, serogroup *Hebdomadis* serovar *Hebdomadis*, serogroup *Icterohaemorrhagiae*, *Copenhageni*, serovar *Icterohaemorrhagiae*, serogroup *Louisiana* serovar *Louisiana*, serogroup *Pomona* serovar *Pomona*, serogroup *Pyrogenes* serovar *Zanoni*, serogroup *Sejroe Hardjo* serovar *Balcanica*, *Saxkoebing*, *Sejroe*, and serogroup *Tarassovi* serovar *Tarassovi*.

Screening was performed using a 1:100 dilution of serum due to the endemic nature of the disease. Agglutination against a 1:100 dilution of serum was considered a positive result, and two-fold serial dilutions titrated the sample to determine the highest positive titer. The presumed infecting serogroup was the serogroup against which the highest agglutination titer was directed [8]. A sample with a high agglutination titer for several serogroups was defined as mixed.

### 2.5. Criteria for Defining Confirmed Leptospirosis

A sample was considered positive for leptospirosis in the MAT when a four-fold increase in titer to one or more serovars was present between the parallel-mounted acute and convalescent-phase serum samples or if the titers were equal to or greater than 1:800 with compatible symptoms [5].

### 2.6. Seroprevalent Leptospirosis

In negative cases of human leptospirosis, a titer of ≥1:100 was found against one or more serovars in the MAT. There was no change in titer between the samples in the acute and convalescent phases, which indicated a previous exposure to *Leptospira* spp.

### 2.7. Information Processing and Statistical Treatment of Data

The data were tabulated in a Microsoft Excel spreadsheet. Statistical analyses were performed using the IBM SPSS statistical software version 25.0. Kappa coefficients [29], positive and negative predictive values, sensitivities, and specificities were calculated. The online tool MICE (Modeling Center for Infectious Diseases, Mahidol-Oxford Research Unit, Thailand http://mice.tropmedres.ac/home.aspx (accessed on 8 June 2023)) was used for Bayesian latent-class modeling [24]. This model made it possible to determine the probability that a specific participant was a carrier of leptospirosis based on the persistence of the disease and the results of a serological test that determined its sensitivity and specificity.

## 3. Results

During the study period, 275 participants with clinical suspicion of leptospirosis were recruited, including 180 males and 95 females; 18.1% were under 12 years of age, 10.18% were between 13 and 18 years of age, 61.45% were between 19 and 60 years of age, and 10.8% were over 60 years of age. Of the 275 participants included in this study, 92.7% presented with fever, headache, and myalgia associated with jaundice and hepatomegaly (28% and 13.5%, respectively). Of these, 13.5% were confirmed positive for leptospirosis. The most frequent symptoms in the confirmed participants were myalgia (100%), headache (96.4%), fever (97.3%), jaundice (62.2%), nausea (45.9%), and abdominal pain (40.5%). Hepatomegaly (22.9%) was also associated with fever, headaches, and myalgia. The least frequent symptoms were conjunctival suffusion (4.4%), lymphadenopathy (3.6%), and hemoptysis (2.5%). In confirmed cases, fever, headache, and myalgia accompanied by jaundice and hepatomegaly occurred in 59.5% and 29.7%, respectively. Table 1 displays the results according to the diagnostic techniques used.

This study included 223 convalescent phase serum samples. Of 52 participants, the second sample was unavailable (convalescence phase), 21 died before the second sample was collected, and the remaining 31 did not visit the medical institution providing health services for the second sample (Table 1). Table 2 lists the sensitivity, specificity, positive predictive values (PPVs), and negative predictive values (NPVs) of diagnostic tests implemented during the acute and convalescent phases using the MAT as the gold standard and the Bayesian model.

Table 3 presents the Kappa values for each technique used in the acute and convalescent phases. Our results showed moderate agreement for the IgM ELISA during the acute phase, while the SD Leptospira and Leptocheck showed slight and fair agreement, respectively. In the convalescent phase, we observed substantial agreements for the IgM-ELISA and fair and slight agreements for Leptocheck and SD Leptospira, respectively.

## 4. Discussion

Various serological tests for diagnosing leptospirosis have been developed and implemented recently; however, their validation has yet to be conducted in Colombia. An ideal diagnostic test should have high sensitivity and specificity during the acute phase, be widely available at a reasonable cost, and provide rapid results. Different sensitivity and specificity values may be obtained depending on the evaluated population and the antigen used. Clinicians must understand these variations in the validation indices of diagnostic tests to assess their effectiveness and reduce misdiagnosis [30] accurately. In the present study, the sensitivity values for the different phases of the disease using the Bayesian model and MAT as the gold standard model ranged from 6.1 to 68.3%. (Table 2). Studies in the Andaman and Nicobar Islands in India [31], Hawaii [23], Thailand [32], and the USA [33] reported sensitivity values between 25 and 92%. These findings differ from those conducted in Sri Lanka, which may be attributed to the higher number of suspected and confirmed participants in this study [14,15].

The results of this study suggest that the MAT is the most effective immunological test for confirming cases in the convalescent phase. Our Bayesian model analysis allowed us to identify true sick participants accurately, revealing a higher confirmation rate in the convalescence phase. However, the MAT showed lower sensitivity in the acute phase (Table 2). These findings are consistent with those of a Sri Lankan study that reported sensitivity values of 55.3% in the acute phase and 95% in the convalescence phase [14]. In contrast, studies from Thailand, Palau, Hawaii, Illinois, and Puerto Rico reported higher sensitivity values in the convalescence phase, likely due to serogroups and serovars with greater circulation in the study regions [33].

Using local serovars in the MAT has been shown to reduce cross-reactivity when local strains are used [34]. For instance, a study in Colombia that included a native strain in the MAT panel found a 15% increase in positivity rates [7]. The antigen panel used in the MAT should include all locally circulating serovars. If these strains are unknown or subject to change, the panel should include serovars representing all applicable serogroups [1]. The sensitivity of the IgM ELISA validated and reported by the manufacturer in Australia and New Zealand was 96.5%. This finding is different from our study and previous reports [35]. Such discrepancies could be due to the unique eco-epidemiological characteristics in each region, which determine disease presentation and behavior. In addition, test values may vary depending on the progression of the disease [8]. While the IgM ELISA showed a higher performance compared to immunochromatographic tests, its sensitivity values were still unsatisfactory. We recommend using immunochromatographic tests as initial screening tools; however, all results must be confirmed using MAT.

The ELISA test demonstrated increased sensitivity in both phases of the infection compared to the MAT, which is considered the gold standard, and the Bayesian model. This improvement can be attributed to a higher production of antibodies observed on day 15 of the disease [8]. Limmathurotsakul et al. [24] concluded that using culture plus the MAT was an imperfect gold standard for comparing diagnostic tests. They found that both sensitivity and specificity improved when using the Bayesian latent class modeling. In leptospirosis, the sensitivity of screening tests can vary depending on the prevalence of different infectious serogroups, which affects their overall performance. All screening tests for leptospirosis need to utilize broadly reactive antigens with various infectious *Leptospira* serovars. The characteristics of serovar panels may vary from one laboratory to another. Therefore, screening tests must be designed to detect antibodies produced against region-specific leptospiral serovars. Some tests use crude antigens derived from *Leptospira biflexa*, serovar Patoc, strain Patoc, and *Leptospira interrogans*, serovar Copenhageni, and strain Wijnberg. Others may use a combination of different *Leptospira* serovars or lipopolysaccharide extracts from various strains of *Leptospira.* Additionally, the amount of antigen applied can influence the intensity of the reaction, resulting in subjective interpretation by the reader [5]. It is essential for laboratories to validate the performance of these tests in the specific setting where they will be used [36].

In contrast to IgM ELISA, the immunochromatographic test SD Leptospira, which detects IgM, showed low specificity in both the acute and convalescent phases of leptospirosis. The Leptocheck WB performed better, exhibiting similar specificities in both phases. This result may be attributed to the persistence of IgM antibodies in confirmed cases of leptospirosis, which can last for months or years [19,37,38]. The findings from this study suggest that immunochromatographic tests do not meet the necessary criteria for screening in areas with difficult geographic access and limited resources because they failed to identify all positive cases. Factors affecting the sensitivity and specificity of these tests could be due to the genus-specific nature of the antigen and their inability to recognize and react to the infecting serovar [15].

The negative predictive values found in the current study for the different tests ranged from 83.5% for SD Leptospira to 93.7% for IgM ELISA. This indicates the likelihood of not having the disease when the test result is negative, which is consistent with findings from previous studies [14,36,39]. Regarding the percentage of concordance or Kappa value (Table 3), it was moderate for IgM ELISA in the acute phase and considerable in the convalescence phase. These results contrast with a study conducted in Brazil [40], which reported concordance for immunochromatographic tests in both phases of the disease. For SD Leptospira testing, the immunochromatographic tests were rated as moderate, and acceptable for Leptocheck. Furthermore, Bathia et al. [9] reported low concordance between the IgM ELISA and LeptoCheck tests in India.

An ideal diagnostic test should exhibit high sensitivity and specificity during the acute phase, be widely accessible at a reasonable cost, and provide rapid and accurate results. Our results showed that the ELISA test outperformed immunochromatographic tests. Although the sensitivity values of ELISA were not acceptable, it may still function as a screening test, provided that all results are confirmed by the MAT. The current study found that a significant percentage (21.62%) of participants had negative IgM ELISA results in both the acute and convalescent phases, later confirmed by the MAT. However, the INS leptospirosis surveillance and control protocol guidelines do not mandate MAT confirmation in cases with negative IgM ELISA results.

Consequently, it is strongly suggested to update these guidelines. Given that MATs can be used for a broader range of serovars, a greater antibody response is expected. In contrast, the results from IgM ELISA may vary depending on the specific antigen used. In Colombia, two imported tests are utilized: Panbio and Virion-Serión. Both assays are designed to detect human IgM antibodies. The Panbio test includes serovars *Hardjo*, *Pomona*, *Copenhageni*, *Australis*, *Madanesis*, *Kremastos*, *Nokolaevo*, *Celledoni*, *Canicola*, *Grippotyphosa*, *Szwajizak*, *Djasiman*, and *Tarassovi*. In contrast, the Virion-Serión test includes non-pathogenic serovars. Using the Virion-Serión IgM ELISA, seropositivity of 39% was reported in Colombia, whereas the MAT showed only 0.3% seropositivity [41]. Overall, the seroprevalence of *Leptospira* in humans in Colombia ranges from 6% to 35%, depending on the geographical area [42].

Compared with other studies with fewer paired sera [40,43], our research benefited from a large number of paired sera, which allowed for the more reliable confirmation of disease cases. Although the ELISA method exhibits high specificity, our study identified some limitations due to suboptimal sensitivity values. This leads us to consider alternative diagnostic methods, such as PCR, especially for use during the acute phase of the disease. Additionally, a study comparing the MAT and PCR found that using PCR alongside the MAT resulted in a more timely and accurate diagnosis of leptospirosis in the early days of infection, particularly when paired serum samples were unavailable. This approach helped reduce the number of indeterminate cases and reduce false negatives that often occur when relying solely on the MAT [44]. In our study, the evaluated serological tests primarily detect IgM antibodies, except the MAT, which detects both IgM and IgG antibodies. IgM antibodies are typically associated with acute infections but can remain in circulation for an extended period. This aspect makes our study unique, as we analyzed immune responses in participants during both phases of infection. One requirement to improve the specificity of the MAT is to isolate and characterize circulating serovars present in the study region. Including these serovars in the diagnostic panel could significantly improve its performance.

Additionally, antibodies may not be detected if the specific serovar causing the illness is absent from the test panel or if low titers are present against similar serovars that are not part of the diagnostic panels. Given the prevalence of other febrile diseases in *Leptospira*-endemic areas, it is essential to improve the diagnosis and clinical identification of leptospirosis. This improvement should be implemented at health service institutions and departmental public health laboratories throughout the Caribbean region to ensure the timely and accurate detection of the disease.

## Figures and Tables

**Table 1:** Comparison of different screening tests for detection of anti-*Leptospira* antibodies in human sera.

Screening Test	Frequency				n
	Positives	%	Negatives	%	
**Acute Phase**					
Leptocheck	16	5.81	259	94.19	275
ELISA	29	10.54	246	89.46	275
SD Leptospira	6	2.18	269	97.82	275
MAT	26	9.45	249	90.55	275
**Convalescent Phase**					
Leptocheck	13	5.82	210	94.18	223
ELISA	27	12.10	196	87.90	223
SD Leptospira	2	0.89	221	99.11	223
MAT	30	13.45	193	86.55	223

**Table 2:** Comparison of the diagnostic accuracies of MAT, SD Leptospira, Leptocheck, and IgM ELISA using Bayesian latent class modeling.

Parameters	MAT as Serologic Gold Standard (%) *		Bayesian Latent Class Model (%) **	
	Acute Phase	Convalescent Phase	Acute Phase	Convalescent Phase
**PARTICIPANTS (%)**	13.5 (9.8–18.2)	14.8 (10.5–20.3)	17.9 (12.8–23.8)	17.4 (12.7–23.0)
**MAT**				
Sensitivity	100	100	75.3 (57.8–91.9)	85.3 (69.9–95.1)
Specificity	100	100	100 (100–100)	100 (100–100)
PPV	100	100	100 (100–100)	100 (100–100)
NPV	100	100	94.9 (89.4–98.6)	97.0 (93.3–99.1)
**SD Leptospira**				
Sensitivity	10.8 (3.5–26.4)	6.1 (1.1–21.6)	9.5 (3.2–20.1)	5.7 (1.1–15.7)
Specificity	99.2 (96.7–99.9)	100 (97.5–100)	99.3 (97.5–100)	99.9 (98.6–100)
PPV	66.7 (24.1–94.0)	100 (19.8–100)	72.5 (32.3–99.5)	90.8 (33.2–100)
NPV	87.7 (83.1–91.3)	86.0 (80.5–90.1)	83.5 (77.7–88.4)	83.5 (77.9–88.1)
**LEPTOCHECK**				
Sensitivity	29.7 (16.4–47.2)	30.3 (16.2–48.9)	31.0 (18.7–45.7)	33.5 (20.1–49.5)
Specificity	97.9 (94.9–99.2)	98.4 (95.1–99.6)	99.6 (97.8–100)	99.9 (98.7–100)
PPV	68.8 (41.5–87.9)	76.9 (46.0–93.8)	94.8 (72.0–100)	98.2 (82.3–100)
NPV	90.0 (85.5–93.2)	89.0 (83.8–92.8)	87.0 (81.4–91.5)	87.7 (82.5–91.9)
**ELISA**				
Sensitivity	54.1 (37.1–70.2)	66.7 (48.1–81.4)	55.0 (39.6–69.4)	68.3 (52.4–81.7)
Specificity	96.2 (92.7–98.1)	97.4 (93.6–99.0)	99.2 (96.2–100)	99.7 (97.6–100)
PPV	69.0 (49.0–84.0)	81.5 (61.3–93.0)	93.6 (71.8–100)	98.0 (84.7–100)
NPV	93.1 (89.0–95.8)	94.4 (89.9–97.0)	91.0 (85.9–94.8)	93.7 (89.3–96.7)

* The gold standard model assumed that the MAT was a perfect test (100% sensitivity and 100% specificity; all participants with a positive gold standard test were diseased, and all participants with a negative gold standard test were not diseased). The values shown are the estimated means with 95% confidence intervals. ** The Bayesian latent class model assumed that all tests evaluated were imperfect. The values shown are the estimated median values with 95% confidence intervals.

**Table 3:** Kappa agreement values for each technique were calculated during the acute and convalescent phases.

Screening Test	Kappa Coefficient	
	Acute Phase	Convalescent Phase
IgM ELISA	0.553	0.692
SD Leptospira (IgM).	0.154	0.099
Leptocheck	0.363	0.383

## Data Availability

The data presented in this study are available on request from the corresponding author.
